# What Do Patients with Celiac Disease Miss the Most Since Starting a Gluten-Free Diet? A Multicenter International Online Survey of Patients and Physicians

**DOI:** 10.3390/nu18172853

**Published:** 2026-09-01

**Authors:** Carolina Ciacci, Martina Sciberras, Yvette Gatt, Suneil A. Raju, Carlo Soldaini, David S. Sanders

**Affiliations:** 1 Academic Unit of Gastroenterology, AOU San Giovanni di Dio e Ruggi d’Aragona, University of Salerno, 84131 Salerno, Italy; cciacci@unisa.it; 2 Gastroenterology Unit, Mater Dei Hospital, MSD 2090 Msida, Malta; muscatmartina@gmail.com (M.S.);; 3 Academic Unit of Gastroenterology, Royal Hallamshire Hospital, Sheffield Teaching Hospitals NHS Foundation Trust, Sheffield S10 2JF, UKdavid.sanders1@nhs.net (D.S.S.); 4 Division of Clinical Medicine, School of Medicine and Population Health, University of Sheffield, Sheffield S10 2RX, UK

**Keywords:** celiac disease, gluten-free diet, therapy, quality of life, patients’ perspective

## Abstract

**Background:** The gluten-free diet (GFD) remains the cornerstone of treatment for celiac disease (CeD), yet it is associated with a substantial psychosocial and practical burden. As novel therapies are being developed, understanding patient priorities, and their alignment with physician perceptions, is essential to guide future therapeutic strategies. **Methods:** We conducted a multicenter, cross-sectional online survey of adults with confirmed CeD followed at tertiary referral centers in Italy (Salerno), the UK (Sheffield) and Malta (La Valletta). The questionnaire collected demographic data, a 0–10 visual analogue scale (VAS) assessing self-reported adherence to the GFD, and responses to a single-choice question exploring what participants missed most since starting the GFD. A parallel survey was administered to healthcare providers to assess their perception of patients’ unmet needs. Descriptive statistics and between-groups comparisons were performed. **Results:** A total of 486 patients (74.7% female; mean age 43.2 years; 215 from Italy, 196 from the UK and 75 from Malta) and 134 physicians were included. GFD adherence was high, with a median score of 10. The most frequently reported unmet need among patients was the desire for an unrestricted diet (25.9%), followed by a preference for a medication allowing occasional gluten exposure (16.9%), greater availability of gluten-free options during travel (15.0%), and improved safety when eating out (13.6%). Interest in a lifelong pharmacological alternative to the GFD was limited (9.5%). Despite minor differences in the distribution of responses across centers, the overall pattern of patient priorities was remarkably consistent. Among 134 physicians (76 (56.7% from the United Kingdom, 20 (14.9%) from Malta, and 38 (28.4%) from Italy)), the most frequently perceived patient need was also an unrestricted diet (17.9%), but a higher proportion attributed importance to lifelong pharmacological therapy (16.4%). The distribution of responses differed significantly between patients and physicians (χ^2^ test, *p* < 0.001). **Conclusions:** Patients with CeD across different European healthcare systems report consistent unmet needs driven primarily by lifestyle constraints and the burden of maintaining a strict GFD. Importantly, most patients do not seek complete replacement of the GFD, but rather strategies that provide greater flexibility and protection against occasional gluten exposure. A significant mismatch exists between patient priorities and physician perceptions, with clinicians overestimating the demand for a definitive cure and underestimating the importance of day-to-day disease burden. Future efforts should improve GFD practicality and prevent accidental gluten exposure, while an unrestricted diet remains the ultimate therapeutic goal.

## 1. Introduction

Coeliac disease (CeD) is a chronic immune-mediated enteropathy triggered by dietary gluten in genetically susceptible individuals and characterized by a heterogeneous spectrum of gastrointestinal and extraintestinal manifestations [1,2,3]. A strict lifelong gluten-free diet (GFD) is effective and remains the only established treatment for CeD [2,3,4]. Nevertheless, dietary treatment requires continuous attention to food selection, labelling, preparation and cross-contamination, imposing a substantial practical and psychosocial burden [5,6].

Although the GFD generally improves symptoms, mucosal damage and health-related quality of life, patients may continue to experience limitations in social activities, travelling, and eating outside the home [6,7,8]. Concern about inadvertent gluten exposure may lead to anxiety and hypervigilance, while accidental exposure remains common even among patients reporting good dietary adherence [7,9]. The limited availability and higher cost of gluten-free products may further affect adherence and quality of life [10]. Indeed, patients with CeD have reported a treatment burden comparable with that associated with some chronic conditions requiring intensive medical treatment [5].

CeD is also increasingly recognized as a major public health concern. A global meta-analysis estimated a serological prevalence of approximately 1.4% [11], while a recent multicentre population-screening study of Italian schoolchildren reported an overall CeD prevalence of 1.65%, with local estimates exceeding 2.5%; approximately 60% of affected children had not been diagnosed before screening [12]. More recently, preliminary findings from the ongoing Italian D1CeScreen paediatric population-screening programme indicated that approximately 2.8–2.9% of screened children tested positive for coeliac-specific antibodies [13,14]. Although diagnostic confirmation and final analyses are still ongoing, these findings suggest that the population requiring assessment and potentially lifelong management may be substantially larger than that identified through symptom-based diagnosis alone. Understanding how best to address the dietary, social and therapeutic needs of this growing patient population is therefore increasingly important.

These limitations have stimulated the development of non-dietary therapies, including gluten-degrading enzymes, agents intended to reduce intestinal permeability or prevent gluten presentation, and immune-modulating or tolerance-inducing strategies [15,16]. Most investigational treatments are currently intended to complement rather than replace the GFD, particularly by providing protection against inadvertent gluten exposure. However, the outcomes prioritized in clinical development may not necessarily reflect patients’ everyday needs and preferences.

Whether patients primarily seek protection against accidental gluten exposure, greater social and dietary flexibility, or complete freedom from dietary restrictions remains unclear. Moreover, healthcare professionals’ perceptions of these priorities may not necessarily align with those of patients. We therefore conducted a multicentre survey in Italy, the United Kingdom and Malta, which differ in their healthcare contexts, approaches to CeD follow-up, and support for access to gluten-free foods. The study aimed to identify the primary unmet need reported by patients with CeD across these settings and to compare patient priorities with healthcare professionals’ perceptions.

## 2. Methods

### 2.1. Study Design and Setting

We conducted an international, cross-sectional online survey across three tertiary referral centers for CeD: a university hospital in Salerno (Italy), the National Centre for Coeliac Disease in Sheffield (UK), and a gastroenterology service in Malta. All centers maintain dedicated CeD registries and routinely invite patients to participate in research activities. The study was conducted in accordance with the Declaration of Helsinki. Approval for observational research involving the assessment of dietary habits and quality of life in patients with celiac disease through questionnaires and surveys administered by e-mail was obtained from the Ethics Committee Campania Sud (Opinion No. 14, 28 February 2019). For the present study, the questionnaire was emailed exclusively to patients who had previously provided explicit consent to be contacted for future celiac disease-related research and surveys. The first page of the online questionnaire included an electronic informed consent form describing the study objectives, the voluntary nature of participation, and data confidentiality. Participants could access the questionnaire only after actively confirming their consent by selecting “Yes.”

### 2.2. Participants

Adult patients (≥18 years) with biopsy- or serology-confirmed CeD who were under follow-up at participating centers and had previously consented to be contacted for research purposes were invited to complete an online survey via personalized emails. Participation was voluntary and anonymous. The overall response rate was approximately 50% and was similar across centers. In parallel, a separate online survey was administered to physicians involved in the management of CeD to assess their perceptions of patients’ unmet needs. Notably, none of those who were asked to fill in the questionnaire refused.

### 2.3. Questionnaire

The survey was developed by investigators and administered as an online questionnaire. It was pilot tested in a small group of patients to ensure clarity, comprehensibility and feasibility.

The questionnaire collected demographic data (age, sex and center) and included a 0–10 visual analogue scale (VAS) assessing self-reported adherence to the GFD.

The primary outcome was based on a single-choice question: “What do you miss the most since starting the gluten-free diet?”, with predefined response options derived from previously published qualitative and quantitative studies on treatment burden and food-related quality of life [1,2,3,4]. Response options included: unrestricted diet, occasional gluten intake, improved availability of gluten-free products (locally and during travel), better safety when eating out, pharmacological options allowing occasional or unrestricted gluten intake, psychological support, and increased awareness among healthcare providers and family members.

A single-choice format was deliberately used to require participants to prioritize their most important unmet need, enabling identification of the dominant patient preference and direct comparison with healthcare professionals’ perceptions.

### 2.4. Statistical Analysis

As this was an exploratory survey based on a single multiple-choice question, no formal hypothesis-driven sample-size calculation was performed; all eligible patients were invited to participate.

Responses to the primary single-choice question were treated as nominal categorical data; no composite or numerical score was generated. Each participant contributed one response to one predefined category. Counts and percentages were calculated separately for patients and healthcare professionals, and the overall distributions were compared using a chi-square test. Fisher’s exact test was used for individual category comparisons when expected cell frequencies were small. Descriptive statistics were generated for all variables. Continuous variables are reported as mean ± standard deviation (SD) or median (interquartile range, IQR), as appropriate, and categorical variables as counts and percentages.

Differences in sex distribution between centers were assessed using chi-square tests. Age and GFD adherence VAS scores were compared between centers using the Kruskal–Wallis test, as data were not normally distributed (Shapiro–Wilk test, *p* < 0.001). Post hoc pairwise comparisons were performed using the Mann–Whitney U test with Bonferroni correction. The association between age and GFD adherence VAS score was assessed using Spearman’s correlation coefficient.

Differences in the distribution of responses to the primary question were evaluated using chi-square tests across centers and between patients and physicians.

All tests were two-sided, and a *p*-value < 0.05 was considered statistically significant. Analyses were performed using SPSS version 31.

## 3. Results

### 3.1. Study Population and Demographic Characteristics

A total of 486 patients (215 from Italy, 196 from the UK and 75 from Malta) and 134 physicians completed the survey. Overall, 74.7% of patients were women, with a significant difference in sex distribution across center, ranging from 82% in Salerno to 60% in Malta. The mean age was 43.2 ± 15.4 years, with no significant difference between centers. Table 1 summarizes the population characteristics.

### 3.2. Adherence to Gluten-Free Diet

VAS data on self-reported adherence to the GFD were available for 369 participants (45.6% from Salerno, 100% from Sheffield and La Valletta). Overall, adherence was high, with a median score of 10 (IQR 9.00–10.00) and a mean score of 9.11 ± 1.873; 63.1% of participants reported a score of 10/10 and a further 18.4% reported 9/10. Within the Salerno cohort, patients with and without available VAS data did not differ significantly in age, sex, or primary-response distribution, although selection bias due to unmeasured factors cannot be excluded.

Participants from Salerno and La Valletta reported slightly but significantly higher VAS scores compared with those from Sheffield (9.36 ± 1.33, 9.75 ± 1.14, and 8.74 ± 2.22, respectively; Kruskal–Wallis H(2) = 35.753, *p* < 0.001; post hoc pairwise comparisons with Bonferroni correction: all *p* ≤ 0.036), although all three centers shared a median score of 10.

A weak but statistically significant positive correlation was found between age and GFD adherence score (Spearman’s rho = 0.119, *p* = 0.022).

### 3.3. Patient-Reported Unmet Needs

The distribution of reported unmet needs differed significantly across centers (Table 2). Across all centers, the most frequently reported unmet need was the desire to return to an unrestricted diet (126/486, 25.9%), followed by the preference for a pharmacological option allowing occasional gluten intake (82/486, 16.9%), greater availability of gluten-free options when travelling (73/486, 15.0%), and improved safety when eating out (66/486, 13.6%). Other reported needs included occasional consumption of gluten-containing products (48/486, 9.9%) and improved local availability of gluten-free products (25/486, 5.1%). Interest in a lifelong pharmacological alternative to the GFD was limited (46/486, 9.5%). Less frequently reported needs included greater awareness among family and friends (7/486, 1.4%), psychological support (6/486, 1.2%), greater awareness among healthcare providers (5/486, 1.0%), and no perceived unmet needs (1/486, 0.2%) (Table 2).

No significant association was found between sex and the distribution of responses; in contrast, age group was significantly associated with the pattern of unmet needs (χ^2^ = 60.07, *p* = 0.002). When stratified by age (≤30, 31–45, 46–60, >60 years), the distribution of responses differed across categories: younger participants (≤30) most frequently selected an unrestricted diet, individuals aged 31–45 years more commonly reported restaurant safety, and those aged 46–60 years indicated limitations related to gluten-free availability during travel. Participants aged > 60 years reported fewer overall needs, although unrestricted diet remained the most frequently selected response.

### 3.4. Healthcare Professionals’ Perceptions of Patients’ Unmet Needs

Of the 134 physicians who completed the survey, 76 (56.7%) were from the United Kingdom, 20 (14.9%) from Malta, and 38 (28.4%) from Salerno, Italy.

Table 3 shows that among healthcare providers, the most frequently perceived patient need was also an unrestricted diet (17.9%). A higher proportion of physicians attributed importance to lifelong pharmacological therapy compared with patients (16.4% vs. 9.5%).

## 4. Discussion

In this multicenter survey, adult patients with CeD from three different European healthcare systems shared similar unmet needs. Participants reported high adherence to the GFD, suggesting overall acceptance of dietary management, yet continued to report missing dietary freedom, spontaneity, and safety when eating outside the home. These findings are consistent with previous studies showing that the GFD imposes a substantial psychosocial and practical burden even among adherent patients in remission [5,6,7,8].

The three participating countries represent distinct healthcare contexts. Italy offers one of the most generous reimbursement systems for gluten-free foods and specialized CeD services; the UK NHS provides structured follow-up, although with more limited access to gluten-free prescriptions, whereas Malta guarantees universal healthcare with a monthly voucher-based system for gluten-free products. Despite these differences, the overall pattern of reported needs was broadly consistent across centers. Although center-specific variations were observed, these appeared to reflect differences in emphasis rather than fundamentally distinct priorities. Across all settings, patients consistently prioritized protection from accidental gluten exposure and improved social flexibility, alongside a desire for complete replacement of the GFD, suggesting that unmet needs are largely related to the lifestyle and psychosocial burden of maintaining a strict GFD [6,7,8,9,10]. Notably, our findings are consistent with the priorities identified by the James Lind Alliance Coeliac Disease Priority Setting Partnership, in which “finding a cure” ranked relatively low among research priorities (8th out of 10), while issues related to everyday disease burden, including eating out, were also highlighted [17]. This alignment supports the notion that patients may prioritize improvements in daily disease management and quality of life over definitive therapeutic solutions [17].

A key finding of this study is the limited interest in a lifelong pharmacological alternative to the GFD. Despite the common desire to return to an unrestricted diet, only a small proportion of patients (9.5%) expressed interest in a drug that would allow daily gluten consumption. In contrast, a substantially larger group favored an occasional-use medication to protect against accidental or infrequent gluten exposure, a pattern that was consistent across centers. Most patients therefore do not appear to seek abandonment of the GFD; rather, they aspire to less fear, fewer constraints, and greater flexibility in specific high-risk situations.

Additionally, in our study, a significant discrepancy emerged between patient-reported needs and healthcare providers’ perceptions, with clinicians placing greater emphasis on pharmacological solutions than patients. This difference may reflect a different interpretation of treatment goals, with clinicians more likely to view a “completely unrestricted diet” as achievable through pharmacological strategies. These findings have important implications for ongoing therapeutic development, suggesting that adjunctive strategies may better align with patient priorities than treatments intended to fully replace dietary management.

VAS data showed very high self-reported adherence to the GFD, consistent with recruitment from specialized referral centers [18]. Mean VAS scores were slightly higher in Salerno and La Valletta than in Sheffield, although all three centers had a median of 10, indicating broadly comparable adherence. However, adherence was self-reported and may overestimate true compliance compared with objective measures such as dietary assessment, serology, or gluten immunogenic peptides. The high reported adherence may partly explain why few patients identified psychological support as a major unmet need, although under-recognition of psychological distress cannot be excluded. Although statistically significant, the correlation between age and GFD adherence was weak (rho = 0.119), suggesting a slight tendency for older participants to report higher adherence, though this association is unlikely to be of clinical relevance.

Our study has strengths and limitations, including its cross-sectional design and possible selection bias among patients willing to participate in online surveys. Patients were recruited from tertiary referral centers and therefore may represent a more informed and motivated population compared with the general coeliac population, potentially limiting generalizability.

The single-choice format was selected deliberately because the study aimed to identify each participant’s primary unmet need rather than to compile an exhaustive list of difficulties associated with the gluten-free diet. Although the forced-choice format facilitated the identification of the primary unmet need, it did not capture the coexistence or relative intensity of other relevant patient priorities.

GFD-adherence data were available for only 45.6% of the Salerno cohort, compared with complete availability in Sheffield and Malta. This uneven pattern of missingness may have introduced selection bias and limited the interpretation of between-center adherence comparisons.

Furthermore, another limitation relates to the interpretation of response options. The distinction between a “completely unrestricted diet” and pharmacological therapies may not have been fully appreciated by patients, particularly if the latter were perceived as adjunctive rather than enabling complete dietary freedom. This may have influenced the relative ranking of these options.

Although dietary freedom and the practical burden of maintaining a GFD emerged as common themes across all three centers, the relative distribution of individual unmet needs differed significantly by center, with notable variation in preferences for an unrestricted diet, restaurant safety, and gluten-free availability during travel. Importantly, many patients appear to prioritize protection from accidental exposure and improved social flexibility alongside, rather than instead of, a complete replacement of the gluten-free diet.

## 5. Conclusions

In conclusion, the findings of the present study suggest that future efforts should prioritize improving the practicality of the GFD and developing adjunctive therapies to mitigate accidental gluten exposure. Nevertheless, a truly effective therapy enabling patients to follow a completely unrestricted diet would remain the ultimate goal. Future studies should therefore assess whether emerging therapeutic strategies can effectively address these patient-reported priorities.

## Figures and Tables

**Table 1:** Baseline demographic characteristics of the study population across participating centers. pts = patients; F= female; M= male; SD = standard deviation.

Center	Number of pts	Sex (F/M)	*p* Value Between Centers	Mean Age ± SD	*p* Value Between Centers
Salerno (Italy)	215	177/38		43.52 ± 13.29	
Sheffield (UK)	196	141/55		44.06 ± 18.77	
La Valletta (Malta)	75	45/30		40.11 ± 9.89	
Total	486	363/123	0.001 *	43.21 ± 15.37	0.154 **

* χ^2^ = 17.35, ** Kruskal–Wallis test, *p* = 0.154.

**Table 2:** Celiac patients’ responses to the question: “what do you miss the most since starting a gluten-free diet?

Response	Total (%)	Sex			Centers			
		Female %	Male %	*p* Value	Salerno Total (%)	Sheffield Total (%)	Malta Total (%)	*p* Value
Gluten itself, meaning a completely unrestricted diet *	126 (25.9)	23.7%	32.8%		34 (15.8)	69 (35.2)	23 (30.7)	
A drug that allows me to occasionally eat foods with gluten **	82 (16.9)	18.7%	11.5%		51 (23.7)	25 (12.8)	6 (8.0)	
Greater availability of gluten-free options when traveling	73 (15.0)	16.3%	11.5%		44 (20.5)	23 (11.7)	6 (8.0)	
Better control in restaurants for the preparation of gluten-free food	66 (13.6)	14.6%	10.7%		29 (13.5)	14 (7.1)	23 (30.7)	
Occasionally eating non-gluten-free products	48 (9.9)	8.0%	14.8%		18 (8.4)	20 (10.2)	10 (13.3)	
A drug that allows me to always eat foods with gluten	46 (9.5)	9.4%	9.8%		20 (9.3)	25 (12.8)	1 (1.3)	
Greater availability of gluten-free products where I live	25 (5.1)	5.0%	5.7%		14 (6.5)	8 (4.1)	3 (4.0)	
Greater awareness of celiac disease among my family and friends	7 (1.4%)	1.4%	1.6%		1 (0.5)	5 (2.6)	1 (1.3)	
Psychological support	6 (1.2%)	1.4%	0.8%		2 (0.9)	4 (2.0)	0 (0.0)	
Greater awareness of celiac disease among doctors	5 (1.0%)	1.1%	0.8%		3 (1.4)	1 (0.5)	1 (1.3)	
Nothing, I am fine	1 (0.2%)	0.3%	0.0%		0 (0)	0 (0)	1 (1.3)	
Overall distribution				0.792				<0.001

*p* values refer to χ^2^ tests comparing the overall distribution of responses by sex and study center. “* Completely unrestricted diet” denotes freedom from dietary restrictions without ongoing medication; ** “a drug that allows me to eat foods with gluten” denotes unrestricted gluten consumption dependent on continuous treatment.

**Table 3:** Physicians’ responses to the question: “what do you think celiac patients miss the most since starting a gluten-free diet?

Response	Total (%)	Female %	Male %	*p* Value Physicians vs. Patients
Gluten itself, meaning a completely unrestricted diet	24 (17.9)	9.0%	9.0%	
A drug that allows me to always eat foods with gluten	22 (16,4)	10.4%	6.0%	0.028 *
Better control in restaurants for the preparation of gluten-free food	21 (15.7)	6.7%	9%	
Occasionally eating non-gluten-free products	21 (15.7)	6.75%	8.9%	
A drug that allows me to occasionally eat foods with gluten	18 (13.4)	3.0%	10.4%	
Greater availability of gluten-free options when traveling	13 (9.7)	3.0%	10.4%	
Greater availability of gluten-free products where I live	5 (3.7)	2.2%	1.5%	
Greater awareness of celiac disease among my family and friends	3 (2.2)	1.5%	0.7%	
Psychological support	1 (0.7)	0.7%	0%	
Greater awareness of celiac disease among doctors	4 (3.0)	2.2%	0.7%	
Nothing, I am fine	1 (0.7)	0.7%	0%	
Overall distribution				<0.001 **

* Fisher’s exact test; ** χ^2^ test.

## Data Availability

The original contributions presented in this study are included in the article. Further inquiries can be directed to the corresponding author.
